# Functional human induced hepatocytes (hiHeps) with bile acid synthesis and transport capacities: A novel *in vitro* cholestatic model

**DOI:** 10.1038/srep38694

**Published:** 2016-12-09

**Authors:** Xuan Ni, Yimeng Gao, Zhitao Wu, Leilei Ma, Chen Chen, Le Wang, Yunfei Lin, Lijian Hui, Guoyu Pan

**Affiliations:** 1 Shanghai Institute of Materia Medica, Chinese Academy of Sciences, Shanghai 201203, China; 2 University of Chinese Academy of Sciences, No. 19A Yuquan Road, Beijing, 100049, China; 3 State Key Laboratory of Cell Biology, Institute of Biochemistry and Cell Biology, Shanghai Institutes for Biological Sciences, Chinese Academy of Sciences, Shanghai, 200031, China

## Abstract

Drug-induced cholestasis is a leading cause of drug withdrawal. However, the use of primary human hepatocytes (PHHs), the gold standard for predicting cholestasis *in vitro*, is limited by their high cost and batch-to-batch variability. Mature hepatocyte characteristics have been observed in human induced hepatocytes (hiHeps) derived from human fibroblast transdifferentiation. Here, we evaluated whether hiHeps could biosynthesize and excrete bile acids (BAs) and their potential as PHH alternatives for cholestasis investigations. Quantitative real-time PCR (qRT-PCR) and western blotting indicated that hiHeps highly expressed BA synthases and functional transporters. Liquid chromatography tandem mass spectrometry (LC-MS/MS) showed that hiHeps produced normal intercellular unconjugated BAs but fewer conjugated BAs than human hepatocytes. When incubated with representative cholestatic agents, hiHeps exhibited sensitive drug-induced bile salt export pump (BSEP) dysfunction, and their response to cholestatic agent-mediated cytotoxicity correlated well with that of PHHs (r^2^ = 0.8032). Deoxycholic acid (DCA)-induced hepatotoxicity in hiHeps was verified by elevated aspartate aminotransferase (AST) and γ-glutamyl-transferase (γ-GT) levels. Mitochondrial damage and cell death suggested DCA-induced toxicity in hiHeps, which were attenuated by hepatoprotective drugs, as in PHHs. For the first time, hiHeps were reported to biosynthesize and excrete BAs, which could facilitate predicting cholestatic hepatotoxicity and screening potential therapeutic drugs against cholestasis.

In the process of drug discovery and development, drug-induced cholestasis has gradually emerged as a major cause of drug-induced liver injury (DILI) and has led to drug withdrawal from the market or the termination of candidate compounds^1^. Hepatic transporter dysfunction, especially drug-induced inhibition of the bile salt export pump (BSEP), can lead to the accumulation of bile acids (BAs) in hepatocytes^1^,^2^,^3^, potentially resulting in mitochondrial dysfunction, oxidative stress, and other types of intrahepatic cholestatic hepatotoxicity^4^,^5^.

Because of species differences^6^, primary human hepatocytes (PHHs) are still considered the gold standard *in vitro* model for investigating the risk of cholestasis in pharmaceutical research^7^. These cells contain the full set of BA synthesis and transport systems^8^,^9^, but their limited availability, short lifespan, high cost and batch-to-batch variability limit their use, especially during the drug discovery phase^10^. Other currently available *in vitro* models have been applied to investigate cholestatic liver damage, but multiple limitations also restrict their use. For example, HepG2 is the most extensively characterized hepatic carcinoma cell line, and it has been used to study cholestatic hepatotoxicity^11^; however, HepG2 cells lack BA transport function^10^,^12^. In addition, HepaRG cells have been used to evaluate cholestatic features, but their low BSEP activity and time-consuming differentiation procedure limit their use^13^,^14^. Both human embryonic stem cell- and induced pluripotent stem cell-derived hepatocytes are promising cell models for studying drug hepatotoxicity^15^; however, their high cost and associated complications restrict their application^16^,^17^. To the best of our knowledge, no study has reported the use of stem cell-derived hepatocytes to study cholestatic toxicity to date.

Direct lineage reprogramming is defined as the direct induction of one specialized cell type into another lineage, with avoidance of the intermediate pluripotent state^18^,^19^,^20^. A previous study has reported the generation of highly proliferative human induced hepatocytes (hiHeps) by linear conversion that have characteristic functions of mature hepatocytes, including glycogen accumulation, albumin excretion, cytochrome P450 (CYP) enzymatic activity and biliary drug excretion^20^. The hiHeps were derived from human fibroblasts induced by the three transcription factors: FOXA3, HNF1A and HNF4A. HNF1A and HNF4A have been reported to promote the mRNA expression of BA synthases^21^ and transporters^22^, implying that this novel cell line may have the capacity for BA synthesis/excretion. However, the ability of these cells to biosynthesize and excrete BA and their potential use for the evaluation of cholestatic liver toxicity have not been yet explored. Thus, in this study, we investigated whether hiHeps could be applied to predict the risk of cholestatic liver toxicity as a hepatocyte-like alternative model to PHHs. We first compared the capacity of hiHeps to biosynthesize and excrete BA with that of PHHs. Then, we examined the potential inhibitory effects of 6 representative cholestatic agents on activity and expression of the BA efflux transporter BSEP in hiHeps. BA-induced direct toxicity was also characterized in both hiHeps and PHHs. Finally, the therapeutic activities of representative hepatoprotective drugs against cholestasis were verified in hiHeps.

## Results

### Expression of the enzymes responsible for BA biosynthesis

**Figure 1:**
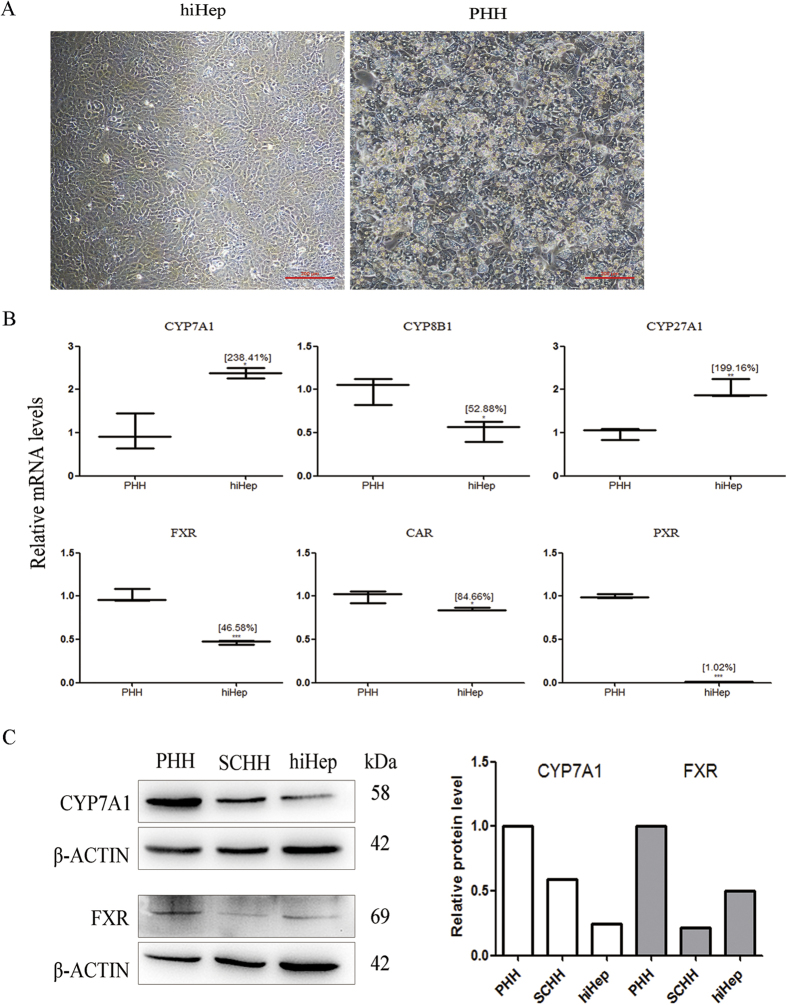
The expression levels of BA synthases in hiHeps and PHHs. (**A**) The typical epithelial morphologies of hiHeps and PHHs, as determined by light phase contrast microscopy. (**B**) The mRNA expression levels of BA synthases (i.e., CYP7A1, CYP8B1, and CYP27 A1) and the upstream nuclear factors (i.e., FXR, CAR, and PXR) in hiHeps. as determined by qRT-PCR. The data are expressed as the mean ± SD (n = 3). *p < 0.05 relative to PHHs. (**C**) Comparison of the protein expression levels of CYP7A1 and FXR among hiHeps, PHHs and SCHHs by western blotting (left) and gray intensity analysis (right). β-ACTIN was used as a reference control.

Under phase-contrast microscopy, cultured hiHeps displayed an epithelial morphology similar to that of PHHs (Fig. 1A). hiHeps expressed the major enzymes responsible for BA synthesis (i.e., cholesterol 7α-hydroxylase (CYP7A1), sterol 12-alpha-hydroxylase (CYP8B1) and sterol 27-hydroxylase (CYP27A1)), as determined by measurement of mRNA levels (238.41%, 52.88% and 199.16% of the levels in PHHs, respectively) (Fig. 1B). The mRNA levels of the nuclear factors farnesoid X receptor (FXR), constitutive androstane receptor (CAR) and pregnane X receptor (PXR) in hiHeps were lower than those in PHHs. In addition, the protein expression of CYP7A1 in hiHeps was 24.44% of that in PHHs and 41.26% of that in sandwich-cultured human hepatocytes (SCHHs) (Fig. 1C). The FXR protein level was close to 50% of that in PHHs (Fig. 1C).

### Expression of the efflux and influx transporters responsible for BA excretion

**Figure 2:**
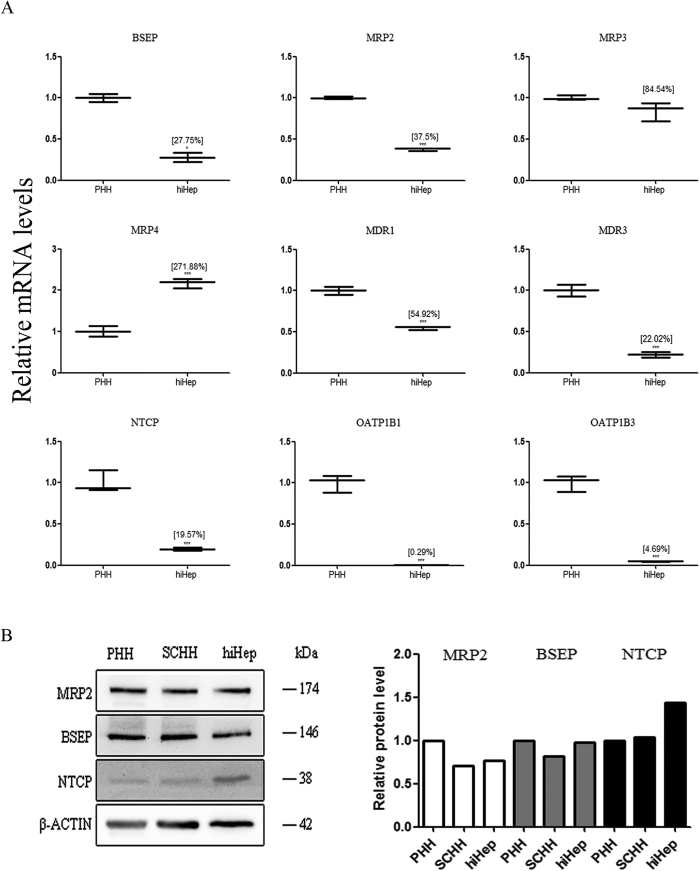
The expression levels of BA transporters in hiHeps and human hepatocytes. (**A**) The mRNA levels of BA efflux (i.e., BSEP, MRPs and MDRs) and influx (i.e., NTCP, OATP1B1 and OATP1B3) transporters in hiHeps determined by qRT-PCR. The data are expressed as the mean ± SD (n = 3). *p < 0.05 relative to PHHs. (**B**) Comparison of the protein expression levels of MRP2, BSEP and NTCP among hiHeps, PHHs and SCHHs by western blotting (left) and gray intensity analysis (right). β-ACTIN was used as a reference control.

**Figure 3:**
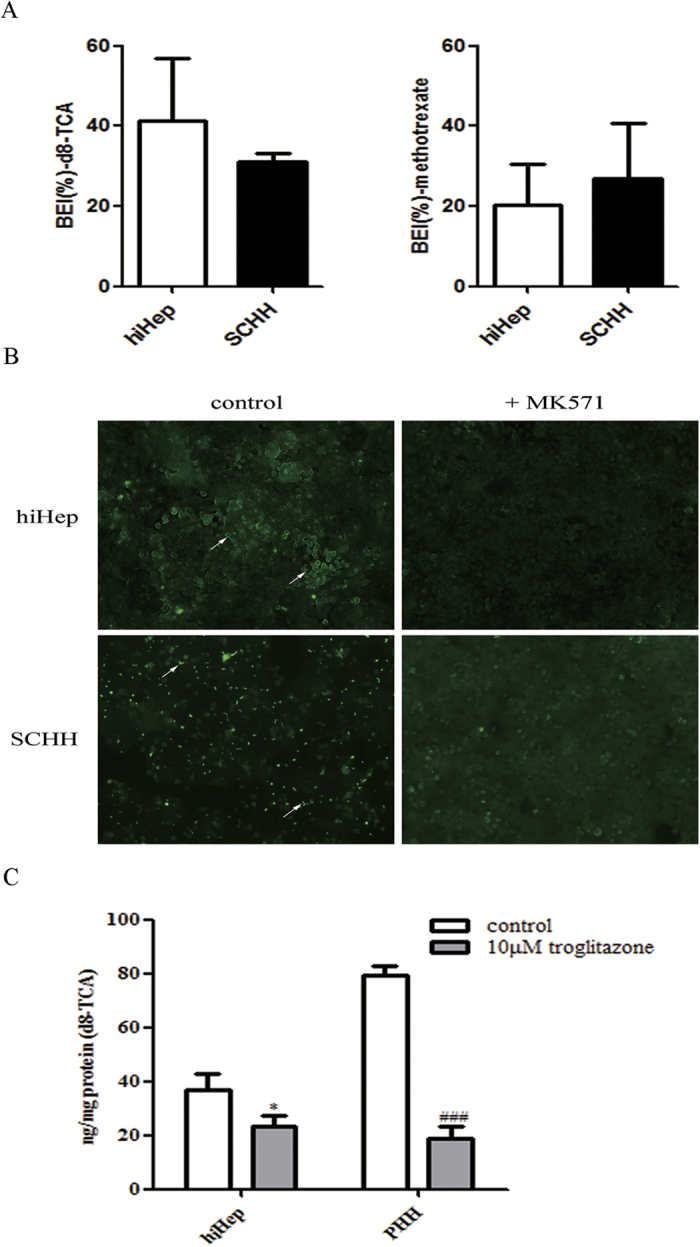
Comparison of the activities of BA transporters between hiHeps and human hepatocytes. (**A**) The efflux transporter activities of BSEP and MRP2 were determined by calculating the BEI values of d8-TCA (the substrate of BSEP) and methotrexate (the substrate of MRP2) for hiHeps (white columns) and SCHHs (black columns). The data are expressed as the mean ± SD (n = 3). (**B**) The polarized locations of efflux transporters in the bile canaliculi of both hiHeps and SCHHs, as determined by fluorescence microscopy. In the absence or presence of MK571 (20 μmol/L), an MRP2 inhibitor, bile canaliculi were labelled with an MRP2 fluorescent substrate, CDF, which was formed from non-fluorescent CDFDA via intracellular esterases. (**C**) The influx transporter activity of NTCP was determined by accumulation assay of an NTCP substrate, d8-TCA. Troglitazone (10 μmol/L) was used as a positive control. The data are expressed as the mean ± SD (n = 3). *p < 0.05 vs control in hiHeps, ^#^p < 0.05 vs control in PHHs.

hiHeps expressed the efflux transporter BSEP (27.75% of the expression in PHHs), multidrug resistance-associated proteins (e.g., MRP2, MRP3 and MRP4, expressed at 37.5%, 84.54% and 271.88% of the levels in PHHs, respectively) and multidrug resistance proteins (e.g., MDR1 and MDR3, expressed at 54.92% and 22.02% of the levels in PHHs, respectively), as determined by measurement of the mRNA levels (Fig. 2A). The mRNA expression related to influx transporters, including the Na(+)-taurocholate cotransport protein (NTCP) and organic anion-transporting polypeptides (e.g., OATP1B1 and OATP1B3), was lower in hiHeps than in PHHs (Fig. 2A). Further, the protein expression of two important efflux transporters BSEP and MRP2 in hiHeps was comparable to that in PHHs and SCHHs (Fig. 2B). The protein level of the influx transporter NTCP was higher in hiHeps than in PHHs and SCHHs (Fig. 2B). Moreover, the biliary excretion index (BEI) values for BSEP and MRP2 in hiHeps were comparable to those in SCHHs (BSEP: 41.36% ± 15.63% vs 31% ± 2.06%; MRP2: 20.17% ± 10.22% vs 26.82% ± 13.72%) (Fig. 3A). Polarization of the efflux transporters was apparent in cultured hiHeps and SCHHs (Fig. 3B). The fluorescent signals in both hiHeps and SCHHs were abolished by an MRP2 inhibitor 5-(3-(2-(7-chloroquinolin-2-yl) ethenyl) phenyl)-8-dimethylcarbamyl-4,6-dithiaoctanoic acid sodium salt hydrate (MK571) (Fig. 3B). The accumulation of deuterium-labelled sodium taurocholate (d8-TCA) in hiHeps was approximately half of that in PHHs. Further, the accumulation of d8-TCA in both cell types was significantly reduced by an NTCP inhibitor, troglitazone (Fig. 3C).

### BA biosynthesis and excretion

**Figure 4:**
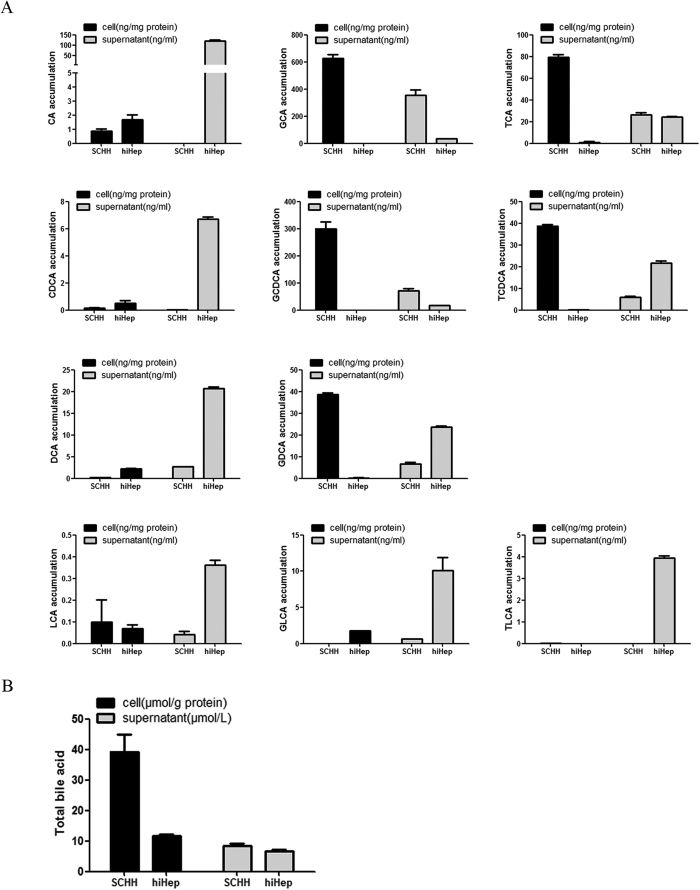
Comparisons of the BA concentrations in cell lysates and supernatants of hiHeps and SCHHs . (**A**) The concentration of each BA (i.e., CA, GCA, TCA, CDCA, GCDCA, TCDCA, DCA, GDCA, LCA, GLCA and TLCA) in both the cell lysates and supernatants of hiHeps and SCHHs was determined by LC-MS/MS. (**B**) The total BA concentrations in the cell lysates and supernatants were measured using a total BA reagent kit.

The concentrations of individual BAs in cell lysates and supernatants were quantitated by liquid chromatography tandem mass spectrometry (LC-MS/MS). The amounts of unconjugated BAs (i.e., cholic acid [CA], chenodeoxycholic acid [CDCA], deoxycholic acid [DCA], and lithocholic acid [LCA]) were comparable between hiHeps and SCHHs (Fig. 4A). Ursodeoxycholate (UDCA) was not detected in either cell type. The concentrations of conjugated BAs (i.e., glycine-conjugated species [GCA, GCDCA, GDCA, and GLCA] and taurine-conjugated species [TCDCA, TCA and TLCA]) were lower in the cell lysate of hiHeps than in that of SCHHs (Fig. 4A). However, the amounts of conjugated BAs (except for GCA, TCA, and GCDCA) were much higher in the supernatant of hiHeps than in that of SCHHs (Fig. 4A). GUDCA, TUDCA and TDCA were not detected in either the cell lysates or the supernatants. The total BA concentration was similar between hiHep and SCHH supernatants but was lower in the hiHep lysate than in the SCHH lysate (Fig. 4B).

### Inhibition of the efflux BA transporter BSEP by representative cholestatic agents

**Figure 5:**
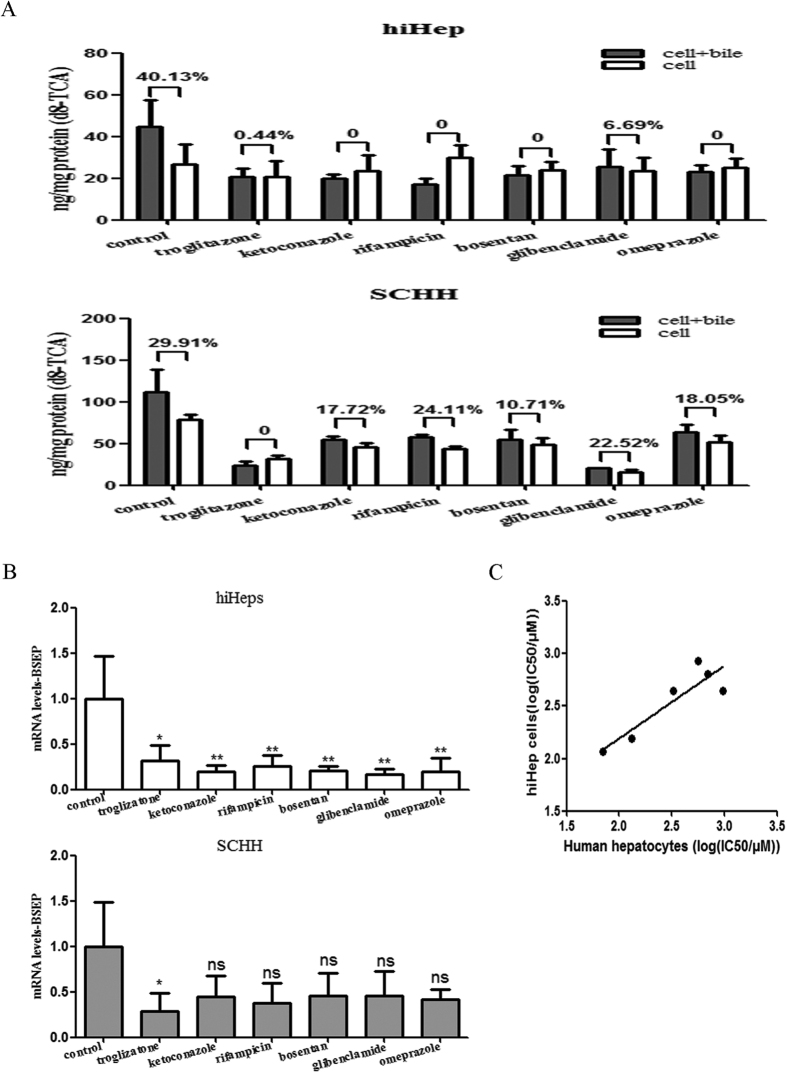
Cholestatic agent-induced BSEP inhibition and cytotoxicity in hiHeps and human hepatocytes. (**A**) BSEP function was inhibited by cholestatic drugs in hiHeps compared with SCHHs, resulting in a decrease in the BEI value of d8-TCA. The cholestatic drugs included 10 μmol/L troglitazone, 30 μmol/L ketoconazole, 25 μmol/L rifampicin, 25 μmol/L bosentan, 10 μmol/L glibenclamide and 100 μmol/L omeprazole. The data are expressed as the mean ± SD (n = 3). (**B**) BSEP expression was inhibited by cholestatic drugs in hiHeps (white columns) compared with SCHHs (grey columns). The data are expressed as the mean ± SD (n = 3). *p < 0.05 vs control. (**C**) Correlation analysis of hiHeps and human hepatocytes based on the log(IC50) determined by MTT assay. The calculated r^2^ value of the linear correlation between hiHeps and human hepatocytes was 0.8032.

The six representative cholestatic agents assessed (troglitazone, ketoconazole, rifampicin, bosentan, glibenclamide and omeprazole) all significantly inhibited BSEP activity following incubation for 15 min, reducing the BEI value from 40.13% to <10% in hiHeps; in contrast, only troglitazone significantly inhibited BSEP activity in SCHHs, reducing the BEI value from 29.91% to <10% at the same concentration (Fig. 5A). In addition to the functional inhibition of BSEP, its mRNA expression was significantly suppressed in hiHeps after incubation with these 6 cholestatic agents. The cholestatic agents also inhibited BSEP mRNA expression in SCHHs, but only troglitazone exhibited significant inhibitory potency (Fig. 5B). Moreover, the responses to the cytotoxicity of these cholestatic agents in hiHeps were positively correlated with those in human hepatocytes (r^2^ = 0.8032) (Fig. 5C).

### BA-induced hepatotoxicity in hiHeps

**Figure 6:**
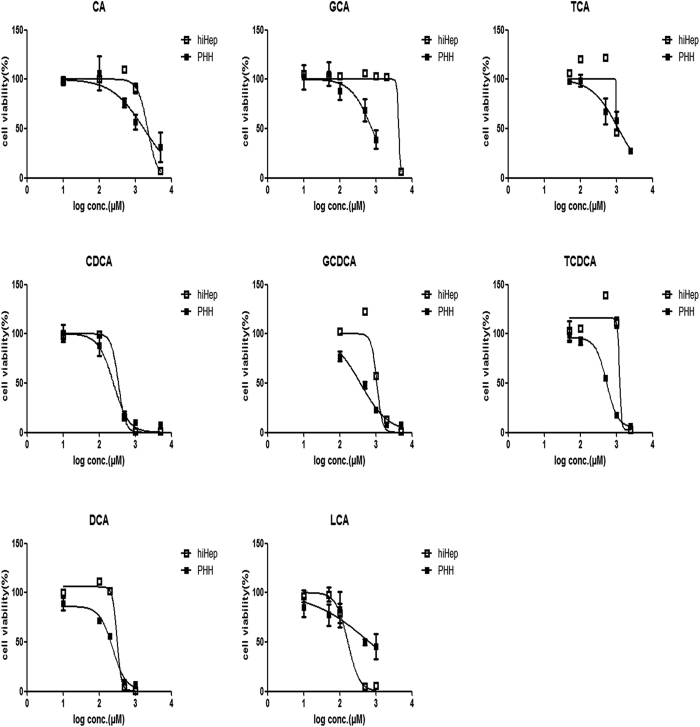
Comparison of the BA concentration-dependent cytotoxicities between hiHeps and PHHs. The BAs included CA, TCA, and GCA (top row); CDCA, TCDCA, and GCDCA (middle row); and DCA and LCA (bottom row). The data are expressed as the mean ± SD (n = 5).

**Figure 7:**
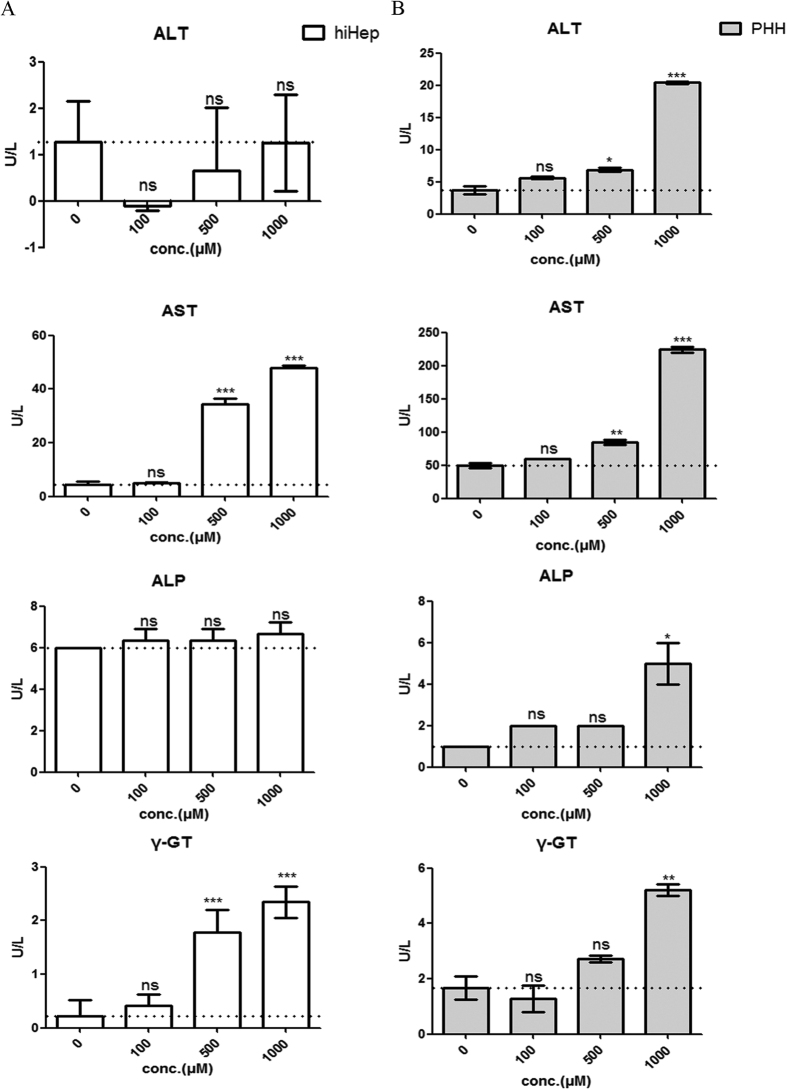
Blood biochemical analysis of DCA-mediated hepatotoxicity in hiHeps and PHHs. (**A**) The enzymatic activities of ALT, AST, ALP and γ-GT in hiHeps. The data are expressed as the mean ± SD (n = 3), *p < 0.05 vs 0 group. (**B**) The enzymatic activities of ALT, AST, ALP and γ-GT in PHHs. The data are expressed as the mean ± SD (n = 2), *p < 0.05 vs 0 group.

The results of 3-(4,5-dimethylthiazol-2-yl)-2,5-diphenyltetrazolium bromide (MTT) assay revealed that the individual BAs decreased the viabilities of hiHeps and PHHs at certain concentrations after 24 hours of incubation (Fig. 6). In contrast to conjugated BAs, the cytotoxicity of the unconjugated BAs (i.e., CA, CDCA, DCA and LCA) was substantially more similar between hiHeps and PHHs (Fig. 6, Table 1). Moreover, the abnormal aspartate aminotransferase (AST) and γ-glutamyl transferase (γ-GT) levels suggested concentration-dependent hepatotoxicity in hiHeps and PHHs after 24 hours of incubation with DCA. The alkaline phosphatase (ALP) level did not obviously change in hiHeps, whereas in PHHs, only treatment with 1000 μmol/L DCA resulted in a slight increase in ALP activity. The alanine aminotransferase (ALT) level did not change in hiHeps, even in the presence of 1000 μmol/L DCA, whereas the ALT activity was significantly increased in PHHs in the presence of 500 or 1000 μmol/L DCA (Fig. 7A and B).

### Cytoprotective effects of representative hepatoprotective agents in hiHeps vs PHHs

**Figure 8:**
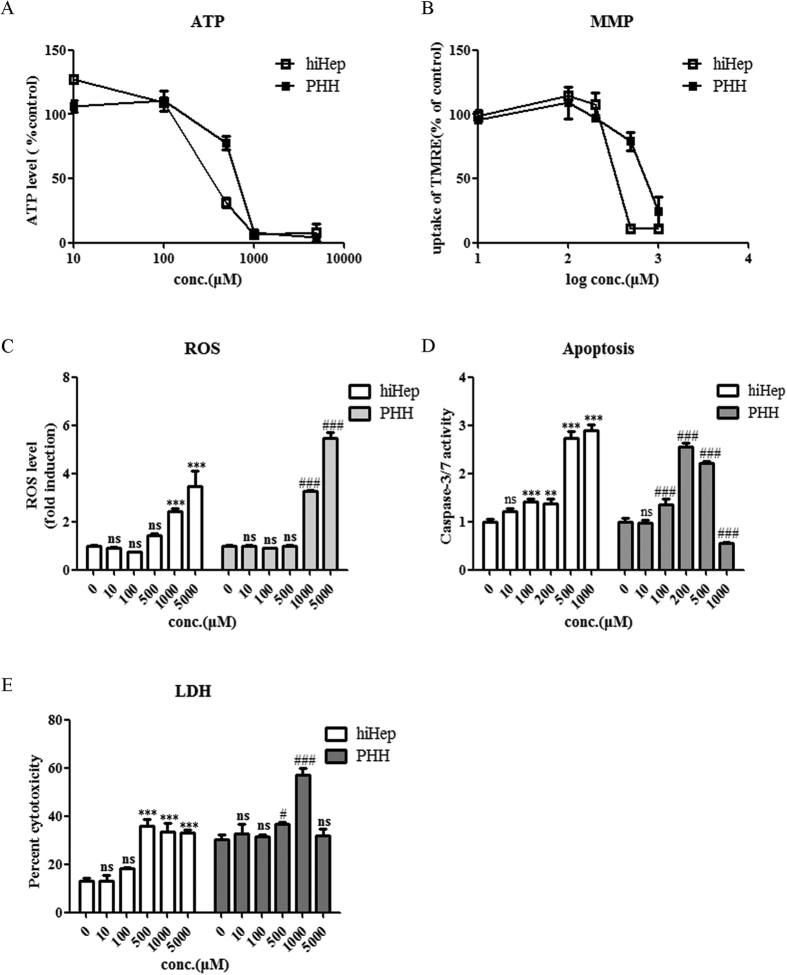
The mechanisms of DCA-mediated toxicity in hiHeps and PHHs. The mechanism of DCA-mediated toxicity was examined by performing ATP (**A**), MMP (**B**), ROS (**C**), apoptosis (**D**) and LDH (**E**) assays. The data are expressed as the mean ± SD (n = 3–5). *p < 0.05 in hiHeps, ^#^p < 0.05 in PHHs.

**Figure 9:**
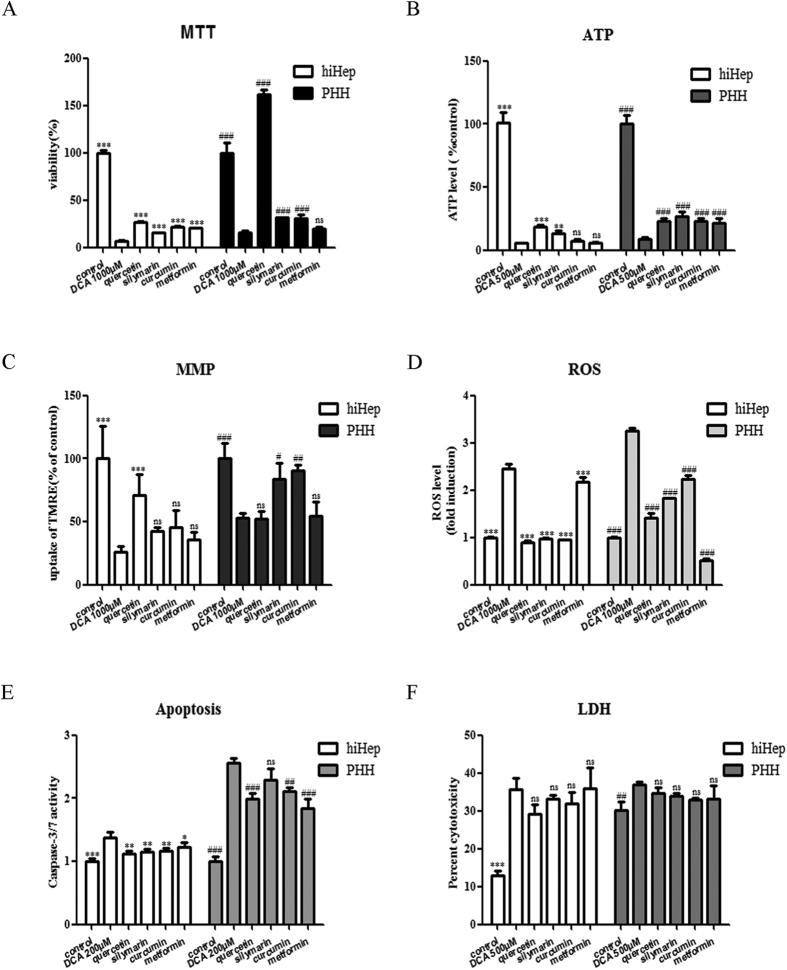
Hepatoprotective effects of therapeutic drugs in hiHeps and PHHs. The hepatic roles of the hepatoprotective drugs quercetin (40 μmol/L), silymarin (25 μmol/L), curcumin (15 μmol/L) and metformin (200 μmol/L) in hiHeps and PHHs were elucidated via MTT (**A**), ATP (**B**), MMP (**C**), ROS (**D**), apoptosis (**E**) and LDH (**F**) assays. The data are expressed as the mean ± SD (n = 3–5). *p < 0.05 vs DCA group in hiHeps, ^#^p < 0.05 vs DCA group in PHHs.

Treatment of hiHeps and PHHs with DCA at certain concentrations exposure resulted in decreases in cell viability (Fig. 6), the ATP concentration (Fig. 8A), and the mitochondrial membrane potential (MMP) (Fig. 8B) and increases in the production of reactive oxygen species (ROS) (Fig. 8C), caspase 3/7 activity (Fig. 8D) and lactate dehydrogenase (LDH) release (Fig. 8E). Co-incubation with representative hepatoprotective drugs (i.e., quercetin, silymarin, curcumin and metformin) for 24 h protected hiHeps and PHHs against DCA-induced decreases in cell viability and the ATP level (Fig. 9A and B). The MMP was enhanced by quercetin in hiHeps, whereas it was significantly increased by silymarin and curcumin in PHHs (Fig. 9C). All four of these compounds significantly reduced DCA-induced ROS production and apoptosis (Fig. 9D and E), but they did not prevent the DCA-induced LDH release in hiHeps or PHHs (Fig. 9F).

## Discussion

BAs play significant roles in the digestion of lipids, nutrients and vitamins and the regulation of cholesterol homeostasis^23^,^24^,^25^,^26^. The biosynthesis of BAs from cholesterol in the liver is mainly mediated by CYP7A1 (the rate-limiting enzyme for BA synthesis), CYP8B1 and CYP27A1^27^,^28^. The transcriptional activation of BA synthases is primarily mediated by nuclear factors, including FXR, PXR and CAR^29^. The excretion of BAs proceeds readily via glycine or taurine conjugation^23^, which is mediated by a broad range of efflux and influx transporters^30^. BA efflux transporters include BSEP, MRPs and MDRs^13^,^31^,^32^. Hepatocellular BA influx is mediated predominantly by NTCP and OATPs^32^. Our study is the first report of the comparable expression of BA synthases and transporters between hiHeps and PHHs (Figs 1 and 2). To compare protein levels and determine the influence of hepatic polarity on protein expression^33^, we used both PHHs and SCHHs as positive controls to assess the protein expression levels of BA synthases and transporters in hiHeps. The BA synthases and efflux and influx transporters normally govern hepatic BA concentration. Thus, we measured the total BA amount in hiHeps and found that the amount in the cell lysate attained to 30% of that in the SCHH lysate. The lower total intercellular BA amount in hiHeps might be attributable to the relatively low CYP7A1 protein level in these cells (Fig. 1C). Interestingly, the CYP7A1 mRNA level was significantly increased in hiHeps compared with PHHs, while the CYP7A1 protein level was significantly decreased. This apparent uncoupling of CYP7A1 mRNA and protein levels might be due to different mechanisms for the transcriptional and translational regulation of the gene and protein expression. The precise reason for this difference needs to be further explored. In addition, CYP7A1 expression is regulated by FXR, and it is decreased when the FXR level is increased^34^. Our results showed that reduced FXR mRNA expression was correlated with increased CYP7A1 mRNA expression in hiHeps. However, a reduction in the FXR protein level was not correlated with an increase in the CYP7A1 protein level in these cells. The possible reason for this finding was that the CYP7A1 protein level was still lower in hiHeps than in SCHHs following FXR-mediated negative regulation. However, the total BA amount in the hiHep supernatants was similar to that in the SCHH supernatants (Fig. 4B), consistent with the comparable BA efflux transport activities detected between these two cell types (Fig. 3A). Interestingly, the LC-MS/MS data revealed that the levels of unconjugated BAs in the hiHep lysate were comparable to those in the PHH lysate, whereas the levels of conjugated BAs were lower in the hiHep lysate (Fig. 4A). These lower levels of conjugated BAs might have been due to reduced expression of the enzymes (e.g., BA-CoA synthetase [BACS] and BA-CoA: amino acid N-acetyltransferase [BAT]) responsible for BA conjugation (data not shown). Because the serum concentration of unconjugated BAs is significantly elevated during cholestasis^35^, it was speculated that the unconjugated BA concentration in cells might influence hepatotoxicity to a greater extent than the conjugated BA concentration. In this study, we found that the response to unconjugated BA toxicity in hiHeps was more similar to that in PHHs than the response to conjugated BA toxicity (Fig. 6). This consistent toxic response to unconjugated BAs in hiHeps and PHHs implied that hiHeps could serve as an alternative to PHHs for the prediction of potential cholestatic toxicity.

Cholestasis induced by the over-accumulation of BAs is thought to be a chronic condition, ultimately resulting in liver fibrosis and cirrhosis^31^. One of the most important mechanisms of drug-induced cholestasis is BSEP inhibition^36^. In the clinic, some human cholestatic liver diseases are related to BSEP malfunction, including progressive familial intrahepatic cholestasis (PFIC) and benign recurrent intrahepatic cholestasis (BRIC)^1^,^37^. Importantly, in humans, no compensatory mechanism for the loss of BSEP function exists^31^,^38^. Therefore, the assessment of BSEP inhibition by candidate drugs is extremely important during drug discovery and development^36^. In *in vitro* studies, a BEI value of 10% is the recommended cut-off value for compounds with obvious bile clearance potentials^39^. Thus, we used a BEI value of 10% in our study as the cut-off value for evaluating the potential for drug-induced BSEP inhibition. Our results demonstrated that hiHeps significantly reflected the effects of cholestatic drugs on the inhibition of BSEP function (BEI < 10%) and expression (Fig. 5A and B), reflecting direct and indirect mechanisms of transporter dysfunction, respectively^23^. However, only troglitazone significantly reduced BSEP activity (BEI < 10%) and expression in SCHHs. These results indicated that hiHeps were more susceptible than SCHHs to cholestatic agent-mediated inhibition of both BSEP function and expression. With regard to BSEP function, we think that the increased BSEP sensitivity was potentially due to the reduced uptake of d8-TCA (BSEP substrate) by hiHeps, while in terms of BSEP expression, the increased sensitivity was possibly attributed to the lower BSEP expression in these cells. Thus, hiHeps could be utilized to sensitively predict potential drug-induced BSEP inhibition and the consequent cholestasis risk, especially during the early stages of drug discovery.

To further investigate the potential application of hiHeps in cholestatic research, the cytotoxic hydrophobic compound DCA, which causes hepatocyte cell death during intrahepatic cholestasis^40^, was chosen to induce hepatotoxicity in hiHeps. The results showed that the level of AST, but not that of ALT was increased by DCA in hiHeps, whereas the activities of both of these enzymes were increased in PHHs (Fig. 7A and B). The reason for this difference might be that DCA did not alter the ALT gene or protein level in hiHeps, and thus, ALT activity was not affected^41^. However, an increased AST level alone is not enough to confirm BA-induced hepatotoxicity; thus, we analysed other liver-specific markers, such as ALP and γ-GT. γ-GT is an enzyme that is involved in the first step of glutathione catabolism^42^, and it was significantly elevated in a concentration-dependent manner in hiHeps and PHHs. In addition, the ALP level was slightly elevated in hiHeps and was significantly increased in PHHs following exposure to 1000 μmol/L DCA in PHHs, suggesting the presence of a biliary tract disorder. Taken together, these data suggested that DCA induced hepatotoxicity. Additionally, DCA caused mitochondrial damage, and promoted ROS generation and cell death in both hiHeps and PHHs (Fig. 8), as reported previously^40^. However, the caspase 3/7 activity was decreased shown in Fig. 8D at the higher DCA concentration (500 μmol/L) in PHHs. It might be due to the loss of cellular viability that occurred when the DCA concentration reached 500 μmol/L. Similarly, in Fig. 8E, LDH release was reduced at the highest DCA concentration (5000 μmol/L) observed at in PHHs. The reason for this result was not clear but it might be because of DCA’s limited solubility at that concentration in the PHH medium. These above results indicated that hiHeps could be a good alternative to PHHs for the evaluation of cholestatic toxicity.

We further investigated the protective potentials of hepatoprotective drugs in hiHeps. Quercetin, silymarin and curcumin are antioxidants that have been reported to protect hepatocytes against cholestatic liver damage^43^,^44^,^45^. Our study showed that these compounds also protected hiHeps against DCA-mediated cholestatic toxicity (Fig. 9A–E). However, although quercetin has been reported to mitigate the ethanol-induced LDH increase in rat primary hepatocytes^46^, our results indicated that it had no effect on DCA-mediated LDH release in either cell type (Fig. 9F). One potential reason for these discrepant findings could be that the previous study employed ethanol, whereas DCA was used in our study. Metformin, an effective diabetes drug, has been reported to protect rat primary hepatocytes against GCDCA-induced apoptosis rather than necrosis^47^. Our study demonstrated that metformin effectively protected hiHeps and PHHs against DCA-mediated apoptosis (Fig. 9E) but not necrosis, as evaluated by LDH release (Fig. 9F). These results indicated that hiHeps could be used to screen potential anti-cholestatic candidates.

Our study has provided the first evidence that hiHeps not only have the capacity to biosynthesize and excrete BAs but are also sensitive enough to be used in the evaluation of BSEP inhibition for assessing cholestatic drug-induced toxicity. Additionally, hiHeps could be potentially used in comprehensive assessments of the risk of BA-mediated cytotoxicity and the underlying mechanism, and they could also be developed into a potential *in vitro* model for the screening of drug candidates with anti-cholestatic activity.

## Methods

### Experimental design

Initially, we examined the expression of the key enzymes and transporters responsible for BA biosynthesis and excretion at both the mRNA and protein levels. BA efflux transporter activity was assessed by the BEI assay and polarization location, whereas BA influx transporter activity was evaluated by accumulation assay. To comprehensively assess BA biosynthesis and excretion in hiHeps, we measured the BA concentrations in cell lysates and supernatants by LC-MS/MS or using a commercial total BA reagent kit. To further determine whether hiHeps could be used to study the mechanism of cholestasis, we examined the inhibitory potency of 6 representative cholestatic agents against BSEP, a BA efflux transporter, as well as their cytotoxicities, in both hiHeps and PHHs. Then, we assessed the direct toxicity of individual BAs and further explored the representative DCA-induced BA hepatotoxicity. Finally, we assessed the effects of 4 representative hepatoprotective agents on DCA-induced cytotoxicity in hiHeps. At least 3 replicates were performed per experiment for hiHeps, while 2–3 replicates were performed per experiment for PHHs.

### Cell culture

#### hiHeps

hiHeps transdifferentiated from human fibroblasts induced by FOXA3, HNF1A and HNF4A were obtained from Lijian Hui’s lab and were cultured in rat tail collagen-pre-coated dishes according to a previously described protocol^20^. Cells were routinely passaged when they were almost confluent. The cells were cultured on collagen-coated plates at a seeding density of 100,000 cells per mL for approximately four days and were then used for further assays. In contrast, for BA qualification assay, 1 ml of hepatocyte maintenance medium (HMM) used to culture hiHeps in 6-well plates was collected on day 5 after fresh medium had been added to the cells for 24 h; the remaining medium was simultaneously aspirated and then the plates were washed with phosphate-buffered saline (PBS). Both the collected medium and 6-well plates were frozen for further BA measurement by LC-MS/MS.

#### Cryopreserved PHHs

Cryopreserved PHHs were purchased from the Research Institute for Liver Diseases (RILD) (Shanghai) Co. Ltd. and cultured according to an industrial culturing method. Briefly, PHHs were thawed and seeded at a density of 0.70 × 10^6^ viable cells per mL on collagen-coated plates. The cells were allowed to attach overnight, and then the medium was replaced with fresh medium. On day 3, the PHHs were incubated with toxic drugs or used for accumulation assay. SCHHs have been recommended as the most appropriate *in vitro* model to mimic the hepatobiliary secretory process^48^,^49^; thus, we compared hiHeps with SCHHs rather than with PHHs in terms of the activity and polarization localization of the efflux transporters and the BA amounts in the cell lysates and supernatants. PHHs were overlaid with 0.25 mg/mL Matrigel (BD Biosciences, CA, USA) to form SCHHs. To determine the BA concentrations, the medium in which the SCHHs was cultured in 6-well plates was replaced with fresh medium for 24 hours. On day 5, at 24 h after the addition of fresh medium,1 ml of the SCHH medium was collected; the remaining medium was aspirated and then the plates were washed with phosphate-buffered saline (PBS). Both the collected medium and 6-well plates were frozen for further BA quantification by LC-MS/MS.

### Quantitative real-time PCR (qRT-PCR)

hiHeps were harvested at confluence, whereas PHHs were collected on day 3 after plating. Total RNA was extracted from the hepatocytes using TRIzol reagent (Life Technology, CA, USA), and cDNA synthesis was performed via the reverse transcription of 1 μg RNA using a Primescript RT Reagent Kit (Takara, Shiga, Japan). Gene expression levels were quantified using a real-time PCR kit (Qiagen, Hilden, Germany). Primers for CYP7A1, CYP8B1, CYP27A1, FXR, CAR, PXR, BSEP, MRP2, MRP3, MRP4, MDR1, MDR3, NTCP, OATP1B1, OATP1B3, and β-ACTIN were synthesized according to the primer sequences listed in Table 2. β-ACTIN was used as a reference gene. The targeted genes were amplified using the Qiagen Roter Gene Q instrument (Qiagen, Germany).

### Western blot analysis

Total proteins were extracted from hiHeps, PHHs and SCHHs with radio-immunoprecipitation assay (RIPA) lysis buffer (Beyotime, Haimen, China) containing 1 mmol/L phenylmethylsulfonyl fluoride (PMSF) (Beyotime, Haimen, China). Then, the proteins were separated on 8% polyacrylamide gels and electrophoretically transferred to polyvinylidene difluoride (PVDF) membranes (GE-Amersham, USA). Subsequently, the membranes were blocked with Tris-buffered saline containing 5% defatted milk for two hours and incubated with primary antibodies (CYP7A1, 58 kDa, Absci, 1:1000; FXR, 69 kDa, Santa Cruz, 1:200; MRP2, 174 kDa, Proteintech, 1:600; BSEP, 146 kDa, Abcam, 1:1000; and NTCP, 38 kDa, Abcam, 1:1000) overnight at 4 °C. β-ACTIN (1:4000, Santa Cruz) was selected as an internal reference. Next, the membranes were rinsed, incubated with horseradish peroxidase (HRP)-labelled secondary antibodies for 1 h, rinsed again, and visualized by electrochemiluminescence (ECL) (Tanon, Shanghai, China) using a Tanon-5200 automatic chemiluminescence image analysis system (Tanon, Shanghai, China). Gray intensity analysis of the bands was performed by Image J software (National Institute of Health, MD, USA).

### BEI assay

hiHeps or SCHHs were rinsed three times and pre-incubated with warm Hank’s balanced salt solution (HBSS) with (standard buffer) or without calcium (to disrupt cell tight junctions) at 37 °C for 15 min. Then, the HBSS was removed, and the hepatocytes were incubated for 15 min with 5 μmol/L d8-TCA (Martrex, Inc., Minnesota, USA) in the presence or absence of a BSEP inhibitor (i.e., 10 μmol/L troglitazone, 30 μmol/L ketoconazole, 25 μmol/L rifampicin, 25 μmol/L bosentan, 10 μmol/L glibenclamide and 100 μmol/L omeprazole) or 20 μmol/L methotrexate (Sigma-Aldrich, St. Louis, MO) alone in 300 μL of standard buffer. After incubation, the solution was aspirated, and the cells were washed three times with ice-cold PBS and frozen at −80 °C for LC-MS/MS analysis. The BEI, defined as the proportion of accumulated substrates excreted into the bile canaliculi, was calculated using B-CLEAR^®^ technology as follows: BEI = [A_plus_Ca++_ − A_minus_Ca++_]/A_plus_Ca++_ × 100%, where A is the amount of substrate accumulation in the cells, A_plus_Ca++_ is the amount of substrate accumulation in standard buffer-treated cells (cells + bile) and A_minus_Ca++_ is the substrate accumulation in calcium-free buffer-treated cells (hepatocytes)^39^,^50^.

### Functional polarization analysis

hiHeps or SCHHs were washed with standard buffer and pre-incubated for 20 min in the presence or absence of 20 μmol/L MK571 (Sigma-Aldrich, St. Louis, MO), an MRP2 inhibitor, before the addition of 2 μmol/L 5(6)-carboxy-2′, 7′-dichlorofluorescein diacetate (CDFDA) (BD Biosciences, Palo Alto, CA) for 30 min^51^. Fluorescent CDF formed from non-fluorescent CDFDA was visualized in the bile canaliculi for both hiHeps and SCHHs using a fluorescence microscope (Olympus, Japan).

### Accumulation assay

hiHeps or PHHs were rinsed three times and pre-incubated with standard buffer for 15 min at 37 °C. After aspiration of the standard buffer, d8-TCA uptake was initiated by the addition of standard buffer containing 5 μmol/L d8-TCA and incubation for another 15 min. Troglitazone (Sigma-Aldrich, St. Louis, MO; 10 μmol/L) was selected as a positive control for taurocholate accumulation because it inhibits d8-TCA uptake. After incubation, d8-TCA uptake was terminated by washing the cells three times with ice-cold PBS. Subsequently, the samples were frozen at −80 °C for LC-MS/MS analysis.

### LC-MS/MS analysis

The d8-TCA concentration was analysed by LC-MS/MS (LCMS-8030; Shimadzu, Kyoto, Japan) according to a previously described method^52^. The methotrexate concentration was determined in electrospray ionization (ESI) mode with an Inertsil ODS-4 column (100 mm × 2.10 mm, 3 μm, GL Sciences Inc., Tokyo, Japan). The selected reaction monitoring transitions were 454.6.1 m/z > 308.4 m/z for methotrexate and 180.0 m/z > 110.10 m/z for the internal standard (phenacetin). The column temperature was maintained at 40 °C. The mobile phase consisted of acetonitrile (organic phase) and ultrapure water with 0.1% formic acid (aqueous phase). The substrate amounts were normalized to the protein amounts using a bicinchoninic acid (BCA) protein assay kit (Pierce, Rockford, IL).

To simultaneously quantify 15 BA constituents, a Shimadzu LC-20AD HPLC system coupled with an AB Sciex API 4000 triple quadrupole mass spectrometer was used as previously described^53^. The total BA concentration was determined using a total BA reagent kit (Nanjing Jiancheng Bioengineering Institute, Nanjing, China).

### Blood biochemistry

hiHeps were treated with different concentrations (100, 500 and 1000 μmol/L) of DCA at 4 days after plating, whereas PHHs were treated at 3 days after plating according to the manufacturer’s protocol. After 24 h of treatment, the supernatants of hiHeps and PHHs were harvested for biochemical analyses, and the enzymatic activities of ALT, AST, ALP and γ-GT were determined using an Automatic Clinical Analyser (AU5800, Beckman Coulter, Inc., USA).

### Toxicity evaluation

#### MTT assay

First, 96-well plates were seeded with hiHeps (10,000 cells per well) or PHHs (70,000 cells per well). At the indicated time point, the medium was replaced with 100 μl of fresh medium containing five different concentrations of BSEP inhibitors or BA constituents, and the cells were incubated for 24 hours. Cell viability was assessed using 5 mg/mL MTT (Sigma-Aldrich, St. Louis, MO) reagent. In addition, absorbance was measured with a microplate reader (Biotek, Winooski, VT, USA) at 570 nm.

#### ATP assay

The cellular ATP level was determined using a CellTiter-Glo^®^ Luminescent Cell Viability Assay Kit (Promega, Madison, WI, USA). Luminescence was measured with a microplate reader (Biotek, Winooski, VT, USA).

#### MMP measurement

The MMP was evaluated with 1 μmol/L tetramethylrhodamine ethylester (TMRE) (Sigma-Aldrich, St. Louis, MO)^54^. Briefly, after incubation with TMRE for 30 min, the cells were washed once with PBS containing 0.2% bovine serum albumin (BSA), and fluorescence was measured with a microplate reader (Biotek, Winooski, VT, USA) at excitation and emission wavelengths of 549 and 575 nm, respectively.

#### ROS assay

Cellular ROS production was assessed by monitoring the cellular conversion of 10 μmol/L 2′,7′-dichlorofluorescein diacetate (DCFDA) (Sigma-Aldrich, St. Louis, MO) to dichlorofluorescein (DCF)^55^. Fluorescence intensity was measured at excitation and emission wavelengths of 485 and 530 nm, respectively.

#### Apoptosis assay

Apoptosis was determined by measuring caspase 3/7 activity using a Caspase-Glo^®^ 3/7 Assay Kit (Promega, Madison, WI, USA) according to the manufacturer’s instructions. Luminescence was measured with a microplate reader (Biotek, Winooski, VT, USA).

#### LDH release

LDH was measured using a CytoTox-ONETM Homogeneous Membrane Integrity Assay Kit (Promega, Madison, WI, USA). Fluorescence intensity was measured at excitation and emission wavelengths of 560 and 590 nm, respectively. LDH release indicates potential cellular necrosis^56^.

To establish the protective potentials of hepatoprotective drugs in hiHeps, hiHeps and PHHs were co-incubated for 24 hours with 4 hepatoprotective compounds (quercetin (40 μmol/L), silymarin (25 μmol/L), curcumin (15 μmol/L) and metformin (200 μmol/L)) and DCA in different assays at toxic concentrations (*MTT assay*: 1000 μmol/L; *ATP assay*: 500 μmol/L; *MMP measurement*: 1000 μmol/L; *ROS assay*: 1000 μmol/L; *apoptosis assay*: 200 μmol/L; and *LDH release assay*: 500 μmol/L).

### Data analysis

Statistical analysis was performed using GraphPad Prism 5.03 software (GraphPad Software Inc., La Jolla, CA). The data are expressed as the mean ± SD. Differences between two groups were analysed using the t-test. One-way analysis of variance (ANOVA) was performed to determine the statistical significance among groups. In addition, correlation analysis was conducted using a linear correlation method. In all analyses, differences were considered significant at a p value of <0.05.

## Additional Information

**How to cite this article**: Ni, X. *et al*. Functional human induced hepatocytes (hiHeps) with bile acid synthesis and transport capacities: A novel *in vitro* cholestatic model. *Sci. Rep.* **6**, 38694; doi: 10.1038/srep38694 (2016).

**Publisher's note:** Springer Nature remains neutral with regard to jurisdictional claims in published maps and institutional affiliations.

## Figures and Tables

**Table 1:** Comparison of the cytotoxicities of BAs between hiHeps and PHHs based on MTT assay.

BAs	HiHeps (IC50, μM)	PHHs (IC50, μM)
Unconjugated BAs		
CA	2207	1745
CDCA	341.1	249.2
DCA	314.9	237.3
LCA	168.4	679.2
Conjugated BAs		
GCA	4328	775.8
TCA	998.6	1144
GCDCA	1073	374.2
TCDCA	1224	537

**Table 2:** Primer sequences for qRT-PCR analysis.

Gene	Forward (5′-3′)	Reverse(5′-3′)
β-ACTIN	ATCGCTGACAGGATGCAGAA	TAGAGCCACCAATCCACACAG
CYP7A1	AGAAGCATTGACCCGATGGAT	AGCGGTCTTTGAGTTAGAGGA
CYP8B1	TGGCTTTCCGGAAGAATATG	CTTGGTGCTGGCTGAGTGTA
CYP27A1	AAGCGATACCTGGATGGTTG	TGTTGGATGTCGTGTCCACT
FXR	AACCATACTCGCAATACAGCAA	ACAGCTCATCCCCTTTGATCC
CAR	GTGCTCCTGTGCGGAGTAG	ATGGCAGATAGGCAGTTTCCC
PXR	AAGCCCAGTGTCAACGCAG	GGGTCTTCCGGGTGATCTC
BSEP	GTCGGACCTGCATTGTCATTG	ATGTGTGTCTGAGATTCTTGCATT
MRP2	AGCAGCCATAGAGCTGGCCCTT	AGCAAAACCAGGAGCCATGTGCC
MRP3	GGCGTCTATGCTGCTTTAGG	CCTTGGAGAAGCAGTTCAGG
MRP4	ACTGCACCGTGCTAACCATT	CTTCTGCCTTGCCAAGTTGT
MDR1	ATGAAGTTGAATTAGAAAATGCAG	GGAAACTGGAGGTATACTTTCATC
MDR3	TTGATGGGCAGGATATTAGGA	GTTGGCCTCTTTGACAGCTT
NTCP	GTGGCAATCAAGAGTGGTGTC	ACTGGTCCTGGTTCTCATTCC
OATP1B1	TTGGAGGTGTTTTGACTGCTT	ACAAGTGGATAAGGTCGATGTTG
OATP1B3	GTCCAGTCATTGGCTTTGCA	CAACCCAACGAGAGTCCTTAGG
